# Flavonoid Extract from Seed Residues of *Hippophae rhamnoides* ssp. *sinensis* Protects against Alcohol-Induced Intestinal Barrier Dysfunction by Regulating the Nrf2 Pathway

**DOI:** 10.3390/antiox12030562

**Published:** 2023-02-24

**Authors:** Juan Wei, Jinmei Zhao, Tingting Su, Sha Li, Wenjun Sheng, Lidan Feng, Yang Bi

**Affiliations:** College of Food Science and Engineering, Gansu Agricultural University, Lanzhou 730070, China

**Keywords:** sea buckthorn, intestine, phenolic, tight junction, paracellular permeability, oxidative stress, antioxidant defense system

## Abstract

Alcohol has been demonstrated to disrupt intestinal barrier integrity. Some flavonoid compounds that exert antioxidant activity have a protective effect on intestinal barrier function. As an important medicinal and edible plant, sea buckthorn (*Hippophae*) seeds are rich in flavonoids, but their protective effect on the intestinal barrier has not been reported. In our research, 76 kinds of flavonoids were identified in *Hippophae rhamnoides* ssp. *sinensis* seed residue flavonoids (HRSF) by ultra-performance liquid chromatography–tandem mass spectrometry. Kaempferol-3-O-rutinoside, isorhamnetin-3-O-rutinoside, kaempferol-3-O-robinoside-7-O-rhamnoside, isorhamnetin-3-O-2G-rhamnosylrutinoside, quercetin-3-O-rutinoside, (−)-epigallocatechin, and B type of procyanidin were the most abundant substances, accounting for 15.276%, 15.128%, 18.328%, 10.904%, 4.596%, 5.082%, and 10.079% of all identified flavonoids, respectively. Meanwhile, pre-treatment with HRSF was able to prevent alcohol-induced disruption of intestinal barrier integrity through elevating the transepithelial monolayer resistance value, inhibiting the flux of fluorescein isothiocyanate-dextran, and upregulating the mRNA and protein level of TJs (occludin and ZO-1). Furthermore, it was also able to reverse alcohol-induced oxidative stress through suppressing the accumulation of reactive oxygen species and malondialdehyde, improving the glutathione level and superoxide dismutase activity. Finally, the results showed that HRSF pre-treatment effectively elevated the erythroid-related factor 2 mRNA and protein level compared with the alcohol-alone treatment group. Our research was the first to demonstrate that HRSF could prevent alcohol-induced intestinal barrier dysfunction through regulating the Nrf2-mediated pathway in order to attenuate oxidative stress and enhance TJ expression.

## 1. Introduction

Alcoholic beverages are widely consumed all over the world, resulting in numerous gastrointestinal and liver disorders [1]. Most alcohol is absorbed in the proximal small intestine by simple diffusion after oral administration because of its good water solubility, moderate lipid solubility, and small molecular size [2]. Therefore, the intestine is the primary target of alcohol [3]. The intestinal epithelium, which is a continuous monolayer, is the first barrier of the intestine preventing against the invasion of pathogenic antigens and toxins [4]. Tight junctions (TJs) form between epithelial cells to control the selective permeability of the intestinal epithelium [5]. TJs are composed of various molecular components, including transmembrane proteins (claudins and occludin), peripheral membrane proteins (zonulin occludins, ZOs), tricellulin, and junctional adhesion molecules (JAMs) [6]. Studies have identified that alcohol could disrupt the epithelial TJs, leading to abnormal intestinal leakage of bacterial endotoxins and macromolecules [2]. Moreover, cellular oxidative stress is also an important factor in intestinal barrier dysfunction caused by alcohol [7]. A certain range of alcohol (2.5–15%) can increase the permeability of Caco-2 monolayers, which is a typical model of the intestinal barrier, via activating the production of reactive oxygen species (ROS), changing the expression of TJ proteins, and causing oxidation of the microtubule cytoskeleton [8,9].

Flavonoids are important secondary metabolites in plant-based foods, being known for their antioxidant activities [10]. It has been shown that some flavonoids also exert beneficial effects on intestinal epithelial barrier function. Flavonoid-enriched extracts from orange peel could attenuate the alcohol-induced permeability of the Caco-2 cell monolayer by increasing the expression of TJ proteins, including ZO-1, occludin, and claudin 4 [11]. Meanwhile, naringenin, which is the major flavonoid from citrus fruits, was able to modulate TJ protein expression and improve barrier integrity in Caco-2 monolayers, as well as mitigate colitis in mice, by improving the TJ barrier [12,13]. Furthermore, quercetin and morin were also reported to reverse the decreasing of the trans-epithelial electrical resistance (TEER) value and the increasing of membrane permeability that was induced by high-glucose treatment in the Caco-2 cell monolayer [14]. Since natural extracts from plants that are rich in flavonoids generally have antioxidant and intestinal epithelial barrier protective activities, they could be considered as effective strategies to alleviate alcohol-induced intestinal barrier dysfunction.

Sea buckthorn (*Hippophae*), which is a small tree or deciduous shrub, belongs to the Elaeagnaceae family and is mainly distributed in Europe and Asia [15]. China is the largest sea buckthorn producer in the world [16]. As a medicinal and edible plant, all parts of sea buckthorn, including its leaves, berries, and stems, contain abundant phenolic compounds that are mainly composed of phenolic acids and flavonoids [17,18,19]. Notably, flavonoids occupied over 90% of the content of the total phenolic compounds in sea buckthorn berries, including the seeds [18]. Sea buckthorn seeds are rich in flavonoids and are mainly found in the forms of flavonoid glycoside, isorhamnetin-3-O-sophroside-7-O-rhamnosid, isorhamnetin-3-O-rutinoside, and quercetin-3-O-rutinoside, being the most abundant glycosides; meanwhile, querceitin-3-O-glucoside-7-O-rhamnosides; isorhamnetin-3-O-glucoside-7-O-rhamnosides; quercetin-3-O-sophroside-7-Orhamnosides; kaempherol-3-O-sophroside-7-orhamnosides; isorhamnetin-3-O-glucoside; and their free forms including kaempherol, quercetin, and isorhamnetin were also found in significant amounts [20]. Antioxidant effects are the most important function of the flavonoid-enriched seed extract from sea buckthorn; moreover, it also exerts hypolipidaemic, hypoglycaemic, anti-obesity, anti-hypertriglyceridemia, and anti-hypertensive effects [21,22,23,24]. It could be supposed that sea buckthorn seed extract may show an intestinal epithelial barrier protective effect due to its high flavonoid contents and antioxidant activity.

Until now, sea buckthorn seeds have mainly been used to extract seed oil, while the remaining seed residue has not been effectively utilized. A few studies have reported some flavonoid compounds of sea buckthorn seed, but the composition of its flavonoids has not been comprehensively analyzed. Although antioxidant effects are the most important function of sea buckthorn, it is only demonstrated by 2,2′-azino-bis(3-ethylbenzothiazoline-6-sulfonic acid) radical scavenging activity assay [21], with the bioavailability, absorption, and metabolism of seed extract in cells not being taken into consideration. Thus, the antioxidant activity of sea buckthorn seed extract needs to be tested at the cell level. Meanwhile, no research on the intestinal barrier protection effect of sea buckthorn seed flavonoids has been reported until now. There are seven species and eleven subspecies of sea buckthorn that have been recognized worldwide [25]. *Hippophae rhamnoides* ssp. *sinensis* is the wild sea buckthorn subspecies of China with the broadest distribution and maximum production [16]. In this study, flavonoids of *Hippophae rhamnoides* ssp. *sinensis* seed residues were extracted by aqueous ethanol and purified by macroporous resin. The composition and content of *Hippophae rhamnoides* ssp. *sinensis* seed residue flavonoid extract (HRSF) was analyzed by ultra-performance liquid chromatography combined with tandem mass spectrometry equipped with an electrospray ionization (UPLC-ESI-MS/MS) system. Then, the protective effect of HRSF against alcohol-induced intestinal barrier interruption was investigated for the first time by estimating the change of TEER value and flux of fluorescein isothiocyanate (FITC)-dextran in Caco-2 monolayers. Meanwhile, the impact of HRSF on intestinal barrier integrity was also investigated by testing the TJs mRNA and protein level, including ZO-1 and occludin. Moreover, the antioxidant activity of HRSF, which may be responsible for its intestinal protective function, was illustrated by estimating the production of ROS, malondialdehyde (MDA), and glutathione (GSH), as well as the enzyme activity of superoxide dismutase (SOD). Finally, the regulation of the nuclear factor erythroid-related factor 2 (Nrf2) gene, which is the key factor responding to resisting the oxidative system, was explored in order to elucidate the possible protective mechanism of HRSF against alcohol-induced intestinal barrier dysfunction.

## 2. Materials and Methods

### 2.1. Chemicals

Fluorescein isothiocyanate (FITC)-dextran and 2,7-dichlorodihydrofluorescein diacetate (DCFH-DA) was provided by Sigma-Aldrich (St Louis, MO, USA). TRIpure total RNA extraction reagent (Trizol) and Cell Counting Kit-8 (CCK8) were purchased from BioTeke Corpotation (Beijing, China). Assay kits of Whole Cell Lysis (WLA019), MDA (TBA method) (WLA048), GSH (WLA105), SOD (WLA110), rapid preparation for SDS-PAGE gel (WLA013), electrochemilu-minescence (ECL) detection reagents, goat anti-rabbit IgG-HRP antibody, occludin antibody, ZO-1 antibody, Nrf2 antibody, and β-actin antibody were purchased from Wanleibio Co., Ltd. (Shenyang, China). All the standards were purchased from MedChemExpress (Monmouth Junction, NJ, USA).

### 2.2. Materials

Sea buckthorn (*Hippophae rhamnoides* ssp. *sinensis*) seed residues obtained after extracting seed oil were provided by Gansu Gannong Biotechnology Co., Ltd. (Lanzhou, China).

### 2.3. Extraction, Identification, and Quantification of Phenolic Components

The extraction of *Hippophae rhamnoides* ssp. *sinensis* seed residue flavonoids followed our previously published method [19]. Briefly, seed residues were extracted by aqueous ethanol (ethanol/water, 2:1, *v*/*v*) with the assistance of ultra-sonication; the extraction was then freeze-dried. The dried power was purified by AB-8 macroporous resin and freeze-dried again. The product was collected and stored at −20 °C, namely, the *Hippophae rhamnoides* ssp. *sinensis* seed residue flavonoids (HRSF). Its total phenolic content (TPC) was tested according to the Folin–Ciocâlteu method and expressed as milligram gallic acid equivalent per gram of HRSF weight (mg GA equiv./g HRSF); meanwhile, its total flavonoid content (TFC) was estimated by the NaNO_2_-AlCl_3_-NaOH method and expressed as milligram rutin equivalent per gram of HRSF weight (mg RT equiv./g HRSF).

The composition and content of flavonoid compounds in HRSF was analyzed by the UPLC-ESI-MS/MS system (UPLC, ExionLC™ AD; MS, Applied Biosystems 6500 Triple Quadrupole) (SCIEX, Framingham, MA, USA) according to the method of Chen et al. [26]. A total of 196 kinds of flavonoid compounds were detected (Table S1). The conditions were as follows: UPLC: column, Waters ACQUITY UPLC HSS T3 C18 (100 mm × 2.1 mm i.d. 1.8 µm); solvent system, water with 0.05% formic acid (A), acetonitrile with 0.05% formic acid (B). The gradient elution program was set as follows: 0–1 min, 10–20% B; 1–9 min, 20–70% B; 9–12.5 min, 70–95% B; 12.5–13.5 min, 95% B; 13.5–13.6 min, 95–10% B; 13.6–15 min, 10% B. The flow rate was set at 0.35 mL/min, and the temperature was set at 40 °C. The injection volume was 2 μL. ESI-MS/MS: quadrupole-linear ion trap mass spectrometer (QTRAP)^®^ 6500 + LC-MS/MS System, equipped with an ESI Turbo Ion-Spray interface, operating in positive and negative ion mode and controlled by Analyst 1.6.3 software (Sciex). The ESI source operation parameters were as follows: ion source, ESI+/−; source temperature, 550 °C; ion spray voltage: (IS) 5500 V (Positive), −4500 V (Negative); curtain gas (CUR) was set at 35 psi.

Flavonoids were analyzed using scheduled multiple reaction monitoring (MRM). Data acquisitions were performed using Analyst 1.6.3 software (Sciex). Multiquant 3.0.3 software (Sciex) was used to quantify all compounds. The result was expressed as mg/g HRSF.

### 2.4. Cell Culture

Caco-2 cells (Bioleaf Company, Shanghai, China) were cultured in Dulbecco’s modified Eagle’s medium (DMEM) (Servicebio Technology Co., Ltd., Wuhan, China) with 25 mmol/L glucose, 4 mmol/L L-glutamine, 1 mmol/L sodium pyruvate, and phenol red, supplemented with 100 U/mL penicillin, 100 μg/mL streptomycin, and 10% fetal bovine serum (FBS) (Zhejiang Tianhang Biotechnology Co., Ltd., Huzhou, China) at 37 °C with 5% CO_2_.

### 2.5. Analysis of Cell Cytotoxicity

The CCK8 assay was used to analyze the cytotoxicity of HRSF [27]. Caco-2 cells were maintained for 21 days, then treated with HRSF (2.5 μg/mL–320 μg/mL, concentrated as microgram gallic acid equivalent per milliliter) for 24 h. The absorbance was assessed after incubation with CCK8 for 3 h at 450 nm by an iMARK microplate reader (Bio-Rad Laboratories, Inc., Hercules, CA, USA). Cell viability was assessed as the percentage of the mean value normalized to untreated cells.

### 2.6. Measurement of TEER Value and Paracellular Permeability

The TEER assay was applied to estimate intestinal barrier integrity [11,27]. Briefly, Caco-2 cells were planted in the apical chamber of a 12-well transwell plate. After 21 days, cells were pre-treated with HRSF (5, 10 and 20 μg/mL) in serum-free medium for 1 h prior to treatment with 7.5% alcohol for 24 h in a protective group. Meanwhile, wells treated only with alcohol or only with HRSF were used as the alcohol-induced damage group or the HRSF-alone treatment group, respectively. Cells without alcohol and HRSF served as the control group. The medium was removed, the apical chamber and basolateral chamber were filled with 37 °C Hank’s Balanced Salt Solution to wash the cells twice, and then the mixture was incubated at 37 °C with Hank’s Balanced Salt Solution for 30 min. Finally, TEER was estimated with an epithelial volt-ohmmeter ERS-2 (Merck Millipore, Burlington, MA, USA). Resistance values (Ω·cm^2^) were calculated by multiplying the area of the membrane filter. The results were expressed as TEER (% of control) = (TEER_treatment_/TEER_control_) × 100%.

The flux of the FITC-dextran assay was used to estimate the paracellular permeability [27]. Briefly, cells were incubated for 21 days in a transwell plate, then treated as described above. The medium was removed, and cells were washed twice with Hank’s Balanced Salt Solution, as above. The apical chamber was filled with FITC-dextran solution (1 mg/mL), and the basolateral had Hank’s Balanced Salt Solution added; then, the mixture was incubated for 4 h. Finally, the liquid in the basal chamber was collected, and the fluorescence was measured with a SynergyTM HT multi-mode microplate reader (BioTek, Winooski, VT, USA) with an excitation of 490 nm and emission of 520 nm. The content of fluorescence was expressed in relative fluorescence units (RFU). The results were expressed as cumulative transport of FITC-dextran (% control) = (RFU_treatment_/RFU_control_) × 100%.

### 2.7. Quantitative Reverse-Transcription Polymerase Chain Reaction (qRT-PCR) Analysis of mRNA Level

Following the method of Wei et al. [28], respective treatments were given to Caco-2 cells after 21 days culture as described for TEER measurement. The RNA was obtained using TRIpure total RNA extraction reagent (Trizol). Briefly, cells were lysed in 1 mL TRIpure for 5 min, then mixed with 200 μL chloroform and stand still for 3 min. The aqueous phase was mixed with isopropanol, then incubated at −20 °C overnight. The mixture was centrifuged at 4 °C at 10,000× *g*, and the aqueous phase was precipitated with ethanol, whereas total RNA was dissolved with 30 μL RNase-free ddH2O. Then, the first-strand cDNA was synthesized by the PrimeScript RT Reagent Kit, and qRT-PCR was continued by using the SYBR Premix EX TaqTM II kit (Thermo Fisher, Waltham, MA, USA) in an ExicyclerTM 96 fluorescence quantitative instrument (Bioneer Corporation, Daejeon, Republic of Korea). Gene expressions of occludin, ZO-1, and Nrf2 were normalized to β-actin. The results were calculated using the 2^−ΔΔCT^ method. The primers shown in Table S2 were synthesized by the Genscript Biotech Corporation (Nanjing, China).

### 2.8. Western Blot Analysis of Protein Expression

Cells were treated as described in Section 2.6. The whole protein was obtained by the Cell Lysis Kit. Briefly, 600 μL lysis buffer supplemented with 6 μL phenylmethylsulfonyl fluoride (PMSF) was added to cells from one cell culture dish, and then the mixture was centrifuged at 10,000× *g* after incubation on ice for 30 min; the supernatant was the whole cell protein. A total of 40 μg protein of each group was electrophoresed in 10% polyacrylamide gel and transfected onto a polyvinylidene difluoride membrane (Merck Millipore, Burlington, MA, USA). The membrane was blocked in 5% non-fat milk for 1 h and incubated overnight with primary antibodies of occludin, ZO-1, Nrf2, and β-actin at 4 °C. Then, subsequently, it was washed and incubated with goat anti-rabbit IgG-HRP secondary antibody. The protein bands were developed with ECL detection reagents. The optical density was estimated through Gel-Pro-Analyzer software and normalized to β-actin, expressed as fold-over basal change comparative to the control group.

### 2.9. Measurement of ROS

Caco-2 cells were treated as described above. Following this, the cells of each group were collected and stained with DCFH-DA (15 μmol/L) for 20 min. Fluorescence intensity was recorded by flow cytometry (NovoCyte TM, ACEA Biosciences, San Diego, CA, USA) with the excitation of 485 nm and emission of 535 nm. The result was analyzed using NovoExpress software.

### 2.10. Estimation of MDA, GSH Content, and SOD Activity

Cells were cultured and treated as described above, then resuspended with phosphate-buffered saline, lysed ultrasonically in an ice bath, and centrifuged. Protein in the supernatant was quantified with the BCA Protein Assay Kit (WLA004) following the manufacturer’s instructions. Then, the content of MDA and GSH were estimated by the MDA and GSH Assay Kit, expressed as nmol per milligram of cell protein (nmol/mg prot). Meanwhile, the activity of SOD was analyzed by the SOD Assay Kit, expressed as active unit per milligram of cell protein (U/mg prot).

### 2.11. Data Analysis

Results were calculated from the mean ± standard deviation of at least three independent experiments. The statistical analysis was performed with the software of SPSS Statistics 26.0 (IBM, Armonk, NY, USA). The analysis of significant differences was expressed by Duncan’s multiple range tests (*p* < 0.05 means statistically significant difference).

## 3. Results and Discussion

### 3.1. The Flavonoid Composition and Content of HRSF

**Figure 1:**
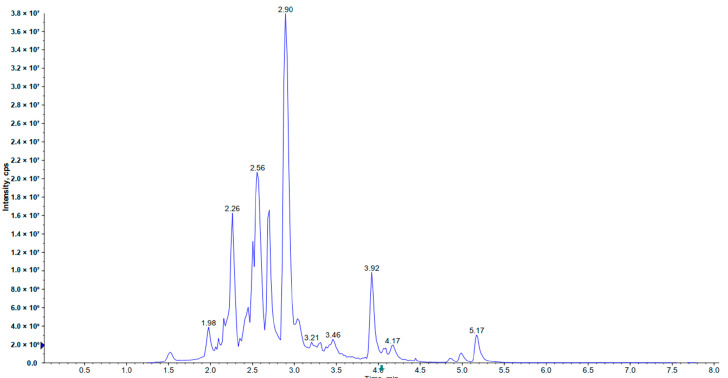
The total ion chromatogram of *Hippophae rhamnoides* ssp. *sinensis* seed residue flavonoids (HRSF).

The results showed that the TPC and TFC of HRSF were 327.759 mg GA equiv./g HRSF and 277.356 mg RT equiv./g HRSF, respectively (Table 1). Thus, it could be inferred that flavonoids were the predominant polyphenols in sea buckthorn seeds, which was also demonstrated through the research of Gong et al. [21]. The HPLC-ESI-MS/MS analysis identified 76 compounds of flavonoids in HRSF, belonging to the subclasses of flavonols, flavanols, procyanidins, flavanones, chalcones, flavanonols, isoflavanones, flavones, and other flavonoids derivatives (Table 1, Figure 1).

#### 3.1.1. Flavonols

A total of 24 compounds of flavonols were identified in *Hippophae rhamnoides* ssp. *sinensis* seed residues; the total content was up to 36.014 mg/g HRSF, which was the highest content subclass of flavonoids, accounting for 73.249% of all identified flavonoids (Table 1, Figure 1). Most of the flavonols in HRSF mainly existed as glycoside forms of kaempferol, isorhamnetin, and quercetin, which was consistent with a previous report by Arimboor and Arumughan [20]. The predominant compounds were robinin (kaempferol-3-O-robinoside-7-O-rhamnoside), nicotiflorin (kaempferol-3-O-rutinosid), and narcissin (isorhamnetin-3-O-rutinoside), up to the content levels of 9.011, 7.511, and 7.438 mg/g HRSF, accounting for 18.328%, 15.276%, and 15.128% of all the identified flavonoids, respectively (Table 1, Figure 1). Arimboor and Arumughan [20] also reported that isorhamnetin-3-O-rutinoside was the major flavonoid of *Hippophae rhamnoides* L. seeds from India. Kaempferol-3-O-rutinosid and kaempferol-3-O-robinoside-7-O-rhamnoside were identified for the first time in sea buckthorn seeds. Typhaneoside (isorhamnetin 3-O-2G-rhamnosylrutinoside) was also the major flavonol glycoside in HRSF, with a concentration of 5.361 mg/g HRSF (Table 1, Figure 1), which was the first time it was identified in sea buckthorn seeds. A significant amount of rutin (quercetin-3-O-rutinoside) was also found in HRSF, up to the content of 2.26 mg/g (Table 1, Figure 1), which was in accordance with a previous report [20]. Meanwhile, kaempferol-3-neohesperidoside, astragalin (kaempferol-3-β-D-glucopyranoside), baimaside (quercetin-3-sophoroside), isorhamnetin-3-O-glucoside, and tiliroside also presented high content levels in *Hippophae rhamnoides* ssp. *sinensis* seeds, at the amounts of 0.570, 0.620, 0.412, 0.525, and 0.178 mg/g HRSF, respectively. Moreover, the free forms of flavonol glycosides, including isorhamnetin, quercetin, kaempferol, and myricetin, were also identified at the levels of 0.726, 0.547, 0.263, and 0.196 mg/g HRSF, respectively. Isorhamnetin, quercetin, and kaempferol were also reported at high levels in 70% methanol extract of *Hippophae rhamnoides* L. seeds from Xinjiang Province, China [29]. However, myricetin was identified for the first time in sea buckthorn seeds. Furthermore, isorhamnetin-3-O-neohespeidoside, quercimeritrin, spiraeoside, afzelin, kaempferitrin, miquelianin, avicularin, laricitrin, 2′′-o-galloylhyperin, and quercitrin were also found in HRSF, but at relatively low levels (less than 0.1 mg/g HRSF).

#### 3.1.2. Procyanidins

Procyanidins are an important subclass of flavonoids in sea buckthorn seed and are only composed of B-type procyanidin [30]. In our research, procyanidin B2 was used as the standard for quantification of the procyanidins—the result showed that the B type of procyanidins presented a significantly high level in sea buckthorn seeds at the level of 4.956 mg/g HRSF and occupied 10.079% of all the identified flavonoids (Table 1, Figure 1).

#### 3.1.3. Flavanols

Flavanol compounds formed the third abundant subclass of flavonoids in *Hippophae rhamnoides* ssp. *sinensis* seeds, accounting for 8.691% of all the identified flavonoids in HRSF (Table 1, Figure 1). (−)-Epigallocatechin was the most abundant compound of this subclass, at the amount of 2.853 mg/g HRSF (Table 1, Figure 1). (−)-Gallocatechin, (−)-catechin, (−)-epicatechin, and (−)-catechin gallate were also present at high levels at 0.763, 0.271, 0.231, and 0.155 mg/g HRSF, respectively (Table 1, Figure 1). Except for (−)-catechin gallate, which was identified firstly in sea buckthorn seed in our research, (−)-epigallocatechin, (−)-gallocatechin, (−)-catechin, and (−)-epicatechin were also reported in a previous research in the water-acetone extract of *Hippophae rhamnoides* ssp. *sinensis* seeds from Shanxi Province, China [31].

#### 3.1.4. Flavanones and Flavanonols

Nine flavanone compounds were found in HRSF, and most of them were in the form of glycosides. Naringenin-7-glucoside, hesperidin, and poncirin were the major flavanones, which were firstly identified in sea buckthorn, presenting at the levels of 0.321, 0.132, and 0.121 mg/g HRSF, respectively. Moreover, isosakuranin, eriodictyol, eriocitrin, pinocembrin, narirutin, and sophoraflavanone G were found at a negligible amount (Table 1, Figure 1). Furthermore, four kinds of flavanonols were firstly found in sea buckthorn seed in our results, namely, dihydrokaempferol, dihydromyricetin, astilbin, and taxifolin 7-O-rhamnoside in the amounts of 0.085, 0.104, 0.062, and 0.087 mg/g HRSF, respectively (Table 1, Figure 1).

#### 3.1.5. Chalcones, Isoflavanones, Flavones, and Other Flavonoids

Six kinds of chalcones were found in HRSF, among them, phlorizin and naringenin chalcone were at high levels at 0.651 and 0.185 mg/g HRSF, respectively. In addition, four kinds of isoflavanones were identified in HRSF as well, and genistin was the highest compound of this subclass, at a concentration of 0.126 mg/g HRSF. Fourteen flavone compounds were also detected, but all at negligible levels. Furthermore, some other kinds of flavonoids were found at a relatively high level, such as mangiferin, theaflavin, and methylnissolin-3-O-glucoside at the concentrations of 0.588, 0.644, and 0.231 mg/g HRSF, respectively.

Generally speaking, flavonols were the predominant subclass of flavonoids in HRSF, and most of them were presented in the form of glycosides. Kaempferol-3-O-rutinoside, isorhamnetin-3-O-rutinoside, kaempferol-3-O-robinoside-7-O-rhamnoside, isorhamnetin-3-O-2G-rhamnosylrutinoside, and quercetin-3-O-rutinoside were the most abundant substances among them. Moreover, (−)-epigallocatechin belonging to the flavone subclass and B type of procyanidin also presented at a significantly high level.

### 3.2. HRSF Alleviated the Alcohol-Induced Decreasing of TEER and Increasing of Paracellular Permeability of the Caco-2 Monolayer

**Figure 2:**
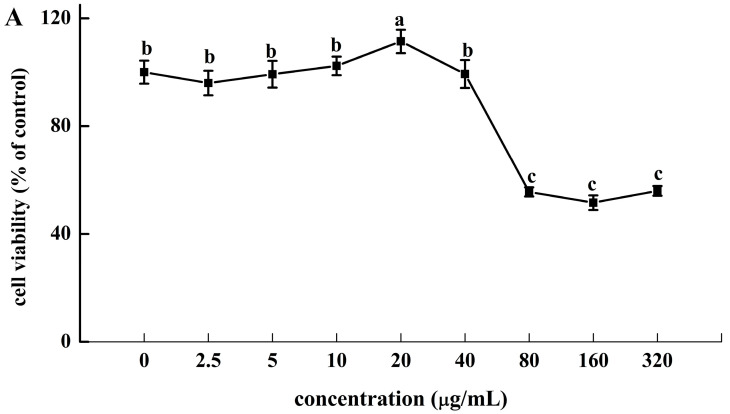

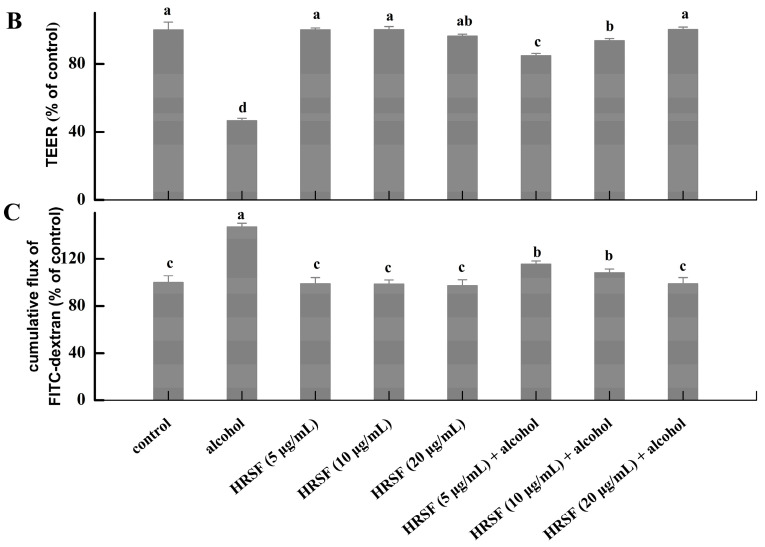
(**A**) Cytotoxicity of *Hippophae rhamnoides* ssp. *sinensis* seed residue flavonoids (HRSF) against Caco-2 cells. (**B**) HRSF restored the alcohol-induced decreasing of TEER value. (**C**) HRSF restored the alcohol-induced increasing cumulative transport of FITC-dextran of the Caco-2 monolayer. Results are expressed as mean ± SD from six replicates for result A, and three replicates for B. Different letters (a, b, c, d) represent significant difference (*p* < 0.05).

HRSF showed no cytotoxicity to Caco-2 cells between 0 and 40 μg/mL. However, it showed significant cytotoxicity above 80 μg/mL, at which cell viability dropped to 55.65% (Figure 2A). Therefore, the maximum concentration of HRSF in subsequent experiments should not exceed 40 μg/mL.

TEER value and paracellular marker FITC-dextran are two common indicators of intestinal barrier integrity and could be used to test membrane permeability [27]. Our results showed that TEER value was decreased by 53.44% and FITC-dextran transportation was increased by 47.24% of alcohol treatment alone, demonstrating that alcohol could cause intestinal barrier dysfunction of the ileum-like Caco-2 monolayer model (Figure 2B,C). Pre-incubation with 5 μg/mL HRSF before treatment with alcohol could increase the TEER by 38.20% and decrease the FITC-dextran diffusion by 31.75% compared with the alcohol alone treatment group. Furthermore, 20 μg/mL HRSF could restore the TEER decrease and FITC-dextran transportation caused by alcohol to the level of control group. It should be noted that the HRSF alone treatment had no significant effect on TEER and FITC-dextran diffusion, and thus we could infer that HRSF significantly prevented the impairments of the intestinal barrier induced by alcohol. This result was consistent with previous finding that some flavonoid extracts or compounds exert protective effects on intestinal barrier integrity against alcohol administration. For example, flavonoid extracts from orange peel prevented alcohol-induced decreasing of the TEER value, as well as the increasing of FITC-dextran diffusion in the Caco-2 monolayer [11]. Luteolin, a dietary flavonoid, also effectively prevents alcohol-induced intestinal barrier injury by increasing TEER value and reducing FITC-dextran transportation [32]. Olejnik et al. [33] reported that blackcurrant fruit extract mainly composed of flavonoid glycosides, such as kaempferol-3-O-rutinosid, isorhamnetin-3-O-rutinoside, and quercetin-3-O-rutinoside, which were also the predominant components in HRSF, could restore the TEER decrease induced by proinflammatory mediators. Thus, we can infer that flavonoid glycosides may play important roles in the intestinal barrier protection effect of HRSF.

### 3.3. HRSF Ameliorated the Alcohol-Induced Downregulation of TJ mRNA and Protein in Caco-2 Cells

The intestinal barrier is formed mainly by TJs, which are multi-protein complexes that link adjacent epithelial cells [34]. Occludin is a tetraspan transmembrane protein, crucial in maintaining the structural integrity and barrier function of TJs [35]. ZO-1 is a cytoskeletal linker protein that provides a link between the transmembrane proteins such as occludin and the cytoskeletal actin [35].

**Figure 3:**
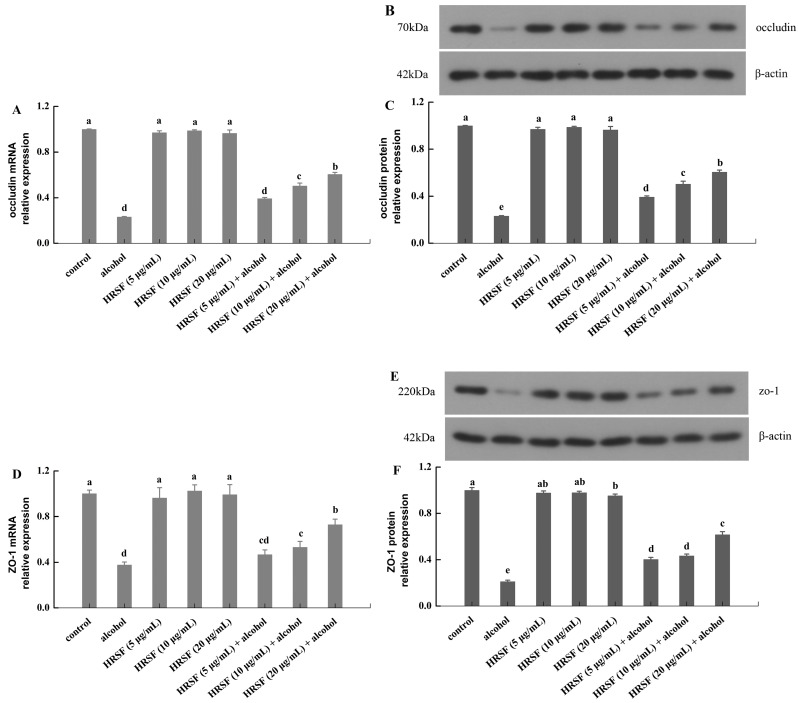
*Hippophae rhamnoides* ssp. *sinensis* seed residue flavonoids (HRSF) ameliorate the alcohol-induced downregulation of occludin and ZO-1 mRNA and protein in Caco-2 cells. The mRNA relative expression of occludin (**A**) and ZO-1 (**D**). The Western blot analysis of occludin (**B**) and ZO-1 (**E**). The protein relative expression of occludin (**C**) and ZO-1 (**F**). The mRNA and protein expression was normalized with the endogenous control gene β-actin and expressed as fold-over basal change comparative to the control group. Results are expressed as mean ± SD from three replicates. Different letters (a, b, c, d, e) represent significant difference (*p* < 0.05).

Our results revealed that alcohol treatment markedly decreased mRNA expression of occludin and ZO-1 by 69% and 62% compared with the control group, respectively (Figure 3A,D). Meanwhile, it also reduced the protein levels of occludin and ZO-1 by 77% and 79%, respectively (Figure 3B,C,E,F). This was also observed in a previous report, finding that 7.5% alcohol could inhibit both the mRNA and protein expression of TJs [11]. Pre-incubation with HRSF prevented the mRNA low-regulation of TJs induced by alcohol in a dose-dependent manner, and 20 μg/mL HRSF increased the mRNA level of occludin and ZO-1 by 2.2- and 1.9-fold, respectively, compared with alcohol treatment alone. In parallel, HRSF have a similar impact on the protein expressions of TJs—20 μg/mL HRSF increased the protein level of occludin and ZO-1 by 2.6- and 2.9-fold in the alcohol group, respectively. Furthermore, individual treatment of HRSF showed no effect on the TJ mRNA and protein level (Figure 3). The above results indicated that HRSF could attenuate the intestinal barrier dysfunction induced by alcohol through upregulating occludin and ZO-1 expression both at transcriptional and translational levels. A similar result was also found in ethanol extract rich in polyphenols from *Alnus japonica* bark, showing a protective effect on the intestinal epithelium in mice with dextran sodium sulfate (DSS)-induced colitis and in HT-29 and Caco-2 cells [36]. Some other flavonoids, such as luteolin, puerarin, and flavonoid-rich propolis extracts, were also found to exert similar effects on occludin and ZO-1, which were downregulated by alcohol [32,37,38]. More importantly, the abundant flavonoids in HRSF, including quercetin-3-O-rutinoside and procyanidin B, have been found to prevent the decreasing of TJ proteins induced by dextran sulfate sodium or inflammatory agents [39,40]. Thus, quercetin-3-O-rutinoside and procyanidin B in HRSF may play key roles in ameliorating the inhibiting effects of alcohol on occludin and ZO-1 expression.

### 3.4. HRSF Attenuated the Alcohol-Induced Generation of ROS and MDA in Caco-2 Cells

**Figure 4:**
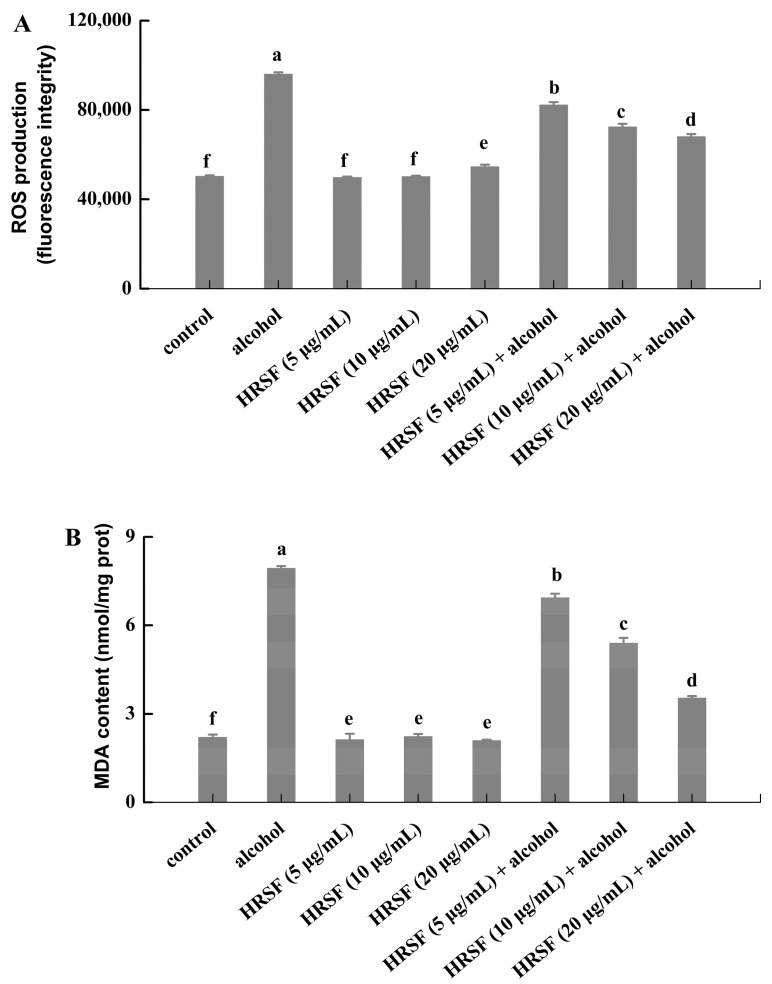
*Hippophae rhamnoides* ssp. *sinensis* seed residue flavonoids (HRSF) attenuate the alcohol-induced generation of ROS and MDA in Caco-2 cells. (**A**) Fluorescence intensity of ROS was recorded with flow cytometry. (**B**) Content of MDA was expressed as nmol per milligram of cell protein (nmol/mg prot). Results are expressed as mean ± SD from three replicates. Different letters (a, b, c, d, e, f) represent significant difference (*p* < 0.05).

The gastrointestinal tract is an important source of ROS production in the body [41]. Although the intestinal epithelial barrier has a protective function, factors such as food, drugs, and exogenous chemicals will also lead to the production of excessive ROS, which will induce oxidative stress in the intestine [41]. Many researchers found that oxidative stress plays a preeminent role in the disruption of the intestinal barrier [42]. Meanwhile, cellular oxidative stress triggered by alcohol was considered to be involved in alcohol-induced intestinal barrier dysfunction [7]. Banan et al. [43] reported that 2.5–15% alcohol could disrupt intestinal barrier integrity and increase paracellular permeability of the Caco-2 monolayer by stimulating excessive production of ROS. The same phenomenon was observed in our results—the total amount of ROS in alcohol-stimulated cells was 1.9 times higher than the control. Furthermore, pretreatment with HRSF was able to significantly reduce the alcohol-induced ROS accumulation in a dose-dependent manner, and HRSF treatment alone had no influence on the ROS production (Figure 4A).

Excessive generation of ROS induced lipid peroxidation, thus causing a large amount of MDA formation, which could react with protein and nucleic acid to make it lose its function [44]. In our results, the MDA production of Caco-2 cell treatment with alcohol was 3.6-fold compared with the control group (Figure 4B), which was in accordance with a previous report that alcohol could promote MDA accumulation [45]. Similar to the effect on ROS production, the MDA content of the HRSF pretreatment group decreased in a dose-dependent manner, reducing to 55.6% in the alcohol alone treatment group at the concentration of 20 μg/mL. Since studies have validated that the accumulation of ROS and MDA could cause intestinal barrier hyperpermeability and disruption [46], the inhibition of ROS and MDA production might partially be the mechanism of HRSF to exert a protective effect on alcohol-induced intestinal barrier dysfunction. Procyanidin B2, which is a major component of HRSF, was reported to attenuate the oxidative stress induced by tert-butyl hydroperoxide in Caco-2 cells through reducing the generation of intracellular ROS [47]. Thus, we could infer that procyanidin B2 plays an important role in the inhibiting effect of HRSF on the alcohol-induced generation of ROS and MDA.

### 3.5. HRSF Restored the Alcohol-Induced Inhibition of the Antioxidant Defense System in Caco-2 Cells

**Figure 5:**
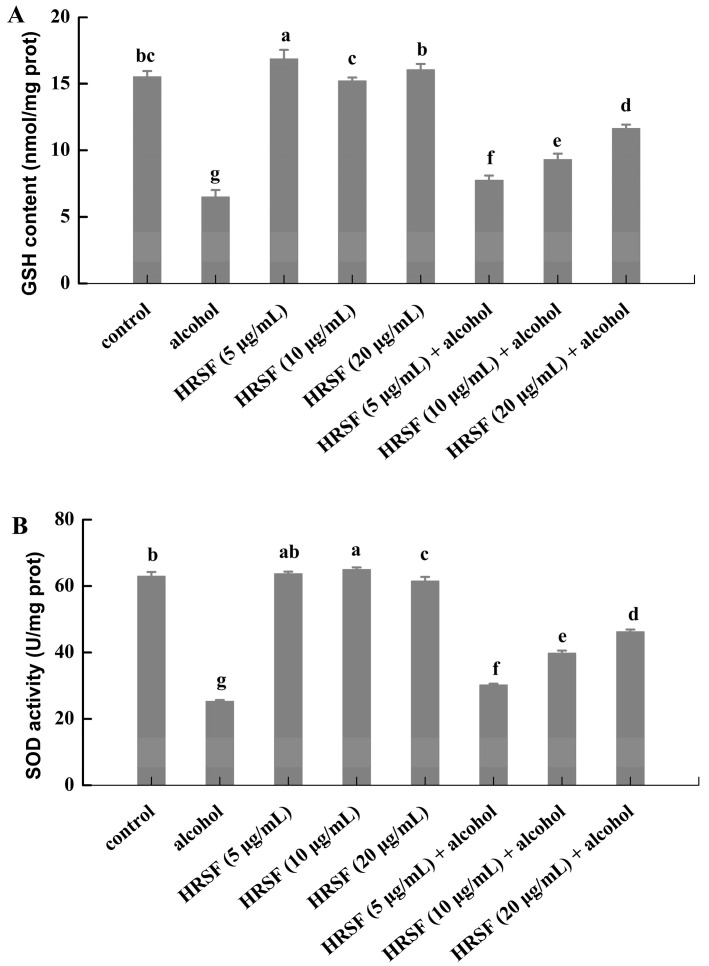
*Hippophae rhamnoides* ssp. *sinensis* seed residue flavonoids (HRSF) restored the alcohol-induced unbalance of oxidative homeostasis by enhancing the GSH content and SOD activity. (**A**) The content of GSH was expressed as nmol per milligram of cell protein (nmol/mg prot). (**B**) The activity of SOD was expressed as active unit per milligram of cell protein (U/mg prot). Results are expressed as mean ± SD from three replicates. Different letters (a, b, c, d, e, f, g) represent significant difference (*p* < 0.05).

The organism has a complex antioxidant defense system that relies on endogenous non-enzymatic and enzymatic antioxidants in response to oxidative stress [48]. GSH is a tripeptide that can directly scavenge ROS without enzymatic help [49]. SOD is the first detoxification enzyme—it catalyzes the dismutation of superoxide anion to O_2_ and H_2_O_2_, which could be removed by the other enzymatic antioxidant systems [50]. Firm evidence has demonstrated that the administration of alcohol could decrease the antioxidant defense level [51]. In our experiment, 7.5% alcohol treatment decreased the level of GSH by 58.1% and suppressed the activity of SOD by 59.9% compared with the control (Figure 5).

Meanwhile, the levels of GSH were increased by 19.1%, 43.2%, and 79.1% in the alcohol treatment group for pre-incubation with 5, 10, and 20 μg/mL HRSF (Figure 5A). In parallel, it also reversed the inhibiting effect of alcohol on SOD activity by 19.5%, 57.4%, and 82.9%, respectively (Figure 5B). However, no significant difference of GSH level and SOD activity was observed between cells challenged exclusively with HRSF and the control cells (Figure 5). Thus, it was obvious that HRSF could restore the redox homeostasis, which was inhibited by alcohol through increasing the GSH content and SOD activity in a dose-dependent manner.

### 3.6. HRSF Prevented Alcohol-Induced Inhibition of Nrf2 in Caco-2 Cells

**Figure 6:**
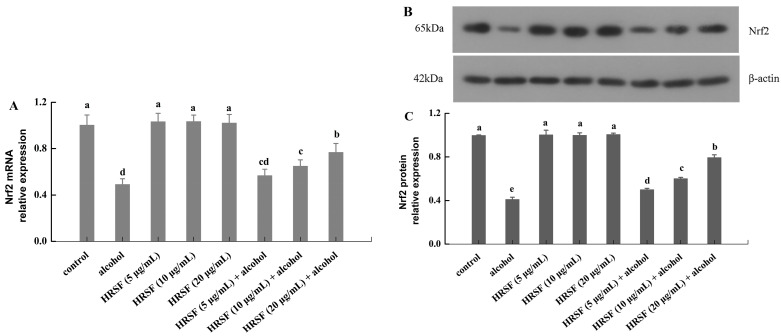
*Hippophae rhamnoides* ssp. *sinensis* seed residue flavonoids (HRSF) prevent alcohol-induced inhibition of Nrf2. The relative mRNA level of Nrf2 (**A**). The Western blot analysis of Nrf2 (**B**). The relative protein level of Nrf2 (**C**). The mRNA and protein expression were normalized with endogenous control gene β-actin and expressed as fold-over basal change comparative to the control group. Results are expressed as mean ± SD from three replicates. Different letters (a, b, c, d, e) represent significant difference (*p* < 0.05).

Nrf2 is a critical nuclear transcription regulator of cells for resisting oxidative stress [52]. It is usually located in the cytoplasm by combining with its negative mediator Keap1 (Kelch-like ECH-associated protein 1) in an inactive state. However, upon stimulation, Nrf2 is activated through dissociating from Keap1 and enters into the nucleus. Then, the activated Nrf2 binds to the antioxidant response element (ARE), which induces the downstream activation of the endogenous antioxidant system [53]. Evidence has shown that Nrf2 plays a key role in maintaining the integrity of the intestinal barrier through alleviating oxidative stress and regulating TJs expression [54]. To elucidate the protection mechanism of HRSF on the intestinal barrier against alcohol, the mRNA and protein levels of Nrf2 were examined. As shown in Figure 6, the mRNA and protein levels of Nrf2 were decreased by 51% and 59% in the alcohol treatment group compared with the control. A previous report also found a similar effect of alcohol on Nrf2 [32]. However, this inhibition induced by alcohol was mitigated by pre-incubation with HRSF in a dose-dependent manner. A 20 μg/mL HRSF pre-incubation before alcohol treatment was able to reverse the mRNA and protein expression of Nrf2 to nearly 80% of the control group. Additionally, the HRSF-alone treatment did not have a significant effect (Figure 6).

Previous studies demonstrated that some flavonoids usually show a protective effect on the intestinal barrier through scavenging of free radicals, but more recent studies indicated that they may also act as indirect antioxidants by triggering the endogenous antioxidant system to balance cellular redox homeostasis [32,55]. Evidence has confirmed that alcohol administration could disrupt the intestinal barrier by stimulating the generation of ROS, suppressing the antioxidant activity and causing the TJ dysfunction [2]. Considering that endogenous antioxidants and TJ expression were regulated by Nrf2 activation [54], and combined with the influence of HRSF on ROS, MDA, GSH content, SOD activity, and TJs expression, we could infer that HRSF could prevent alcohol-induced decreasing of TEER and increasing of paracellular permeability by activating the Nrf2-mediated pathway. Consistent with this, a dietary flavonoid, luteolin, also prevented alcohol-induced intestinal barrier dysfunction by activating the Nrf2-ARE indirect antioxidant system [32].

## 4. Conclusions

In summary, HRSF extracted from sea buckthorn seed residues were mainly com-posed of flavonoids. Flavonols, in the form of glycosides, were the predominant subclass. Kaempferol-3-O-rutinoside, isorhamnetin-3-O-rutinoside, kaempferol-3-O-robinoside-7-O-rhamnoside, isorhamnet-in-3-O-2G-rhamnosylrutinoside, and quercetin-3-O-rutinoside were the most abundant substances among them. Moreover, (−)-epigallocatechin belonging to the flavone subclass and B type of procyanidin were also presented at a significantly high amount. Meanwhile, HRSF had a dramatic protective effect against alcohol-induced intestinal barrier dysfunction, which was mainly achieved through alleviating oxidative stress and enhancing TJ expression. This was combined with the inhibition of ROS and MDA accumulation, the increasing of GSH content and SOD activity, and the activating of Nrf2 induced by HRSF. We could infer that HRSF could prevent alcohol-induced intestinal barrier dysfunction by activating the Nrf2-mediated pathway. Therefore, HRSF may be a useful dietary supplement to prevent intestinal barrier dysfunction induced by alcohol. However, as a mixed flavonoid extract, the main components of HRSF, which play key roles in the intestinal barrier protective effect, as well as their synergistic or suppressive interactions, require further study.

## Figures and Tables

**Table 1:** The composition and content of *Hippophae rhamnoides* ssp. *sinensis* seed residue flavonoids.

Component	Content (mg/g)
Total phenolic content (TPC)	327.759 ± 6.581
Total flavonoid content (TFC)	277.356 ± 16.141
Flavonols	
Narcissin	7.438 ± 0.246
Nicotiflorin	7.511 ± 0.134
Kaempferol-3-neohesperidoside	0.570 ± 0.023
Isorhamnetin-3-O-neohespeidoside	0.036 ± 0.001
Rutin	2.260 ± 0.083
Isorhamnetin	0.726 ± 0.016
Kaempferol	0.263 ± 0.007
Quercimeritrin	0.087 ± 0.008
Quercetin	0.547 ± 0.013
Isorhamnetin-3-O-glucoside	0.525 ± 0.007
Robinin	9.011 ± 0.451
Spiraeoside	0.010 ± 0.001
Afzelin	0.006 ± 0.001
Kaempferitrin	0.019 ± 0.002
Baimaside	0.412 ± 0.014
Tiliroside	0.178 ± 0.006
Astragalin	0.620 ± 0.034
Miquelianin	0.008 ± 0.001
Avicularin	0.020 ± 0.001
Myricetin	0.196 ± 0.013
Laricitrin	0.092 ± 0.003
2″-O-Galloylhyperin	0.021 ± 0.001
Quercitrin	0.098 ± 0.005
Typhaneoside	5.361 ± 0.167
Flavones	
Cynaroside	0.009 ± 0.001
Oroxin A	0.067 ± 0.000
Scutellarin	0.015 ± 0.00
5,7-Dihydroxy-3′,4′,5′-trimethoxyflavone	0.001 ± 0.000
Galangin	0.002 ± 0.000
7,4′-Dihydroxyflavone	0.002 ± 0.000
Luteolin	0.012 ± 0.001
Tricetin	0.102 ± 0.008
Baicalin	0.011 ± 0.001
Pedalitin	0.010 ± 0.001
Apigenin-7-glucoside	0.003 ± 0.000
Chrysin	0.001 ± 0.000
Apigenin	0.005 ± 0.000
Diosmetin	0.002 ± 0.000
Isoflavanones	
Genistein	0.002 ± 0.000
Calycosin	0.001 ± 0.000
Daidzein	0.003 ± 0.000
Genistin	0.126 ± 0.007
Flavanols	
(−)-Epicatechin	0.231 ± 0.007
(−)-Epigallocatechin	2.853 ± 0.193
(−)-Catechin gallate	0.155 ± 0.011
(−)-Catechin	0.271 ± 0.033
(−)-Gallocatechin	0.763 ± 0.028
Anthocyanins	
Procyanidin B2	4.956 ± 0.354
Chalcones	
Phlorizin	0.651 ± 0.080
4′-Hydroxychalcone	0.001 ± 0.000
Trilobatin	0.020 ± 0.001
Benzylideneacetophenone	0.001 ± 0.000
Phloretin	0.002 ± 0.000
Naringenin chalcone	0.185 ± 0.003
Flavanones	
Hesperidin	0.132 ± 0.012
Pinocembrin	0.003 ± 0.000
Sophoraflavanone G	0.001 ± 0.000
Isosakuranin	0.092 ± 0.008
Naringenin-7-glucoside	0.321 ± 0.021
Eriocitrin	0.009 ± 0.002
Eriodictyol	0.091 ± 0.002
Narirutin	0.004 ± 0.000
Poncirin	0.121 ± 0.007
Flavanonols	
Dihydromyricetin	0.104 ± 0.008
Astilbin	0.062 ± 0.002
Taxifolin 7-O-rhamnoside	0.087 ± 0.007
Dihydrokaempferol	0.085 ± 0.002
Flavone glycosides	
Schaftoside	0.009 ± 0.000
Vitexin	0.003 ± 0.000
Orientin	0.009 ± 0.000
Xanthones	
Mangiferin	0.588 ± 0.029
Other flavonoids	
Kurarinone	0.005 ± 0.007
Troxerutin	0.061 ± 0.006
Hydroxysafflor yellow A	0.029 ± 0.003
Theaflavin	0.644 ± 0.097
Methylnissolin-3-O-glucoside	0.231 ± 0.019

Data are presented as mean ± SD, calculated from three biological replicates.

## Data Availability

All the data are available within the article.

## Supplementary Materials

The following supporting information can be downloaded at https://www.mdpi.com/article/10.3390/antiox12030562/s1. Table S1: Information of 196 standard compounds used in the UPLC-ESI-MS/MS method. Table S2: Primer sequence for quantitative reverse transcription polymerase chain reaction.
