# Supplementary material for: Flavonoid Extract from Seed Residues of Hippophae rhamnoides ssp. sinensis Protects against Alcohol-Induced Intestinal Barrier Dysfunction by Regulating the Nrf2 Pathway
Source: Antioxidants (Basel). 2023 Feb 24;12(3):562. doi: 10.3390/antiox12030562 (PMC10044812; doi:10.3390/antiox12030562)
Supplement: Supplementary file 1 [file antioxidants-12-00562-s001.zip › antioxidants-2211060-supplementary-tableS1.pdf]

Supplementary Table S1.

Information of 196 standard compounds used in UPLC-ESI-MS/MS method.

Compounds name	Chemical formula	Molecular Weight	Classification
Isoginkgetin	$C_{32}H_{22}O_{10}$	566.12	Biflavonoids
Ginkgetin	$C_{32}H_{22}O_{10}$	566.12	Biflavonoids
Amentoflavone	$C_{30}H_{18}O_{10}$	538.09	Biflavonoids
Isobavachalcone	$C_{20}H_{20}O_{4}$	324.14	Chalcones
Trilobatin	$C_{21}H_{24}O_{10}$	436.14	Chalcones
Isoliquiritigenin	$C_{15}H_{12}O_{4}$	256.07	Chalcones
Neohesperidin dihydrochalcone	$C_{28}H_{36}O_{15}$	612.21	Chalcones
Sieboldin	$C_{21}H_{24}O_{11}$	452.13	Chalcones
4,4'-Dimethoxychalcone	$C_{17}H_{16}O_{3}$	268.11	Chalcones
4'-Hydroxychalcone	$C_{15}H_{12}O_{2}$	224.08	Chalcones
Phlorizin	$C_{21}H_{24}O_{10}$	436.14	Chalcones
Loureirin B	$C_{18}H_{20}O_{5}$	316.13	Chalcones
Echinatin	$C_{16}H_{14}O_{4}$	270.09	Chalcones
4'-O-Methylbavachalcone	$C_{21}H_{22}O_{4}$	338.15	Chalcones
Benzylideneacetophenone	$C_{15}H_{12}O$	208.09	Chalcones
Naringin Dihydrochalcone	$C_{27}H_{34}O_{14}$	582.19	Chalcones
4-Hydroxychalcone	$C_{15}H_{12}O_{2}$	224.08	Chalcones
Xanthohumol	$C_{21}H_{22}O_{5}$	354.15	Chalcones
Naringenin chalcone	$C_{15}H_{12}O_{5}$	272.07	Chalcones
Licochalcone C	$C_{21}H_{22}O_{4}$	338.15	Chalcones
Licochalcone E	$C_{21}H_{22}O_{4}$	338.15	Chalcones
Phloretin	$C_{15}H_{14}O_{5}$	274.08	Chalcones
(-)-Gallocatechin	$C_{15}H_{14}O_{7}$	306.07	Flavanols
(-)-Catechin gallate	$C_{22}H_{18}O_{10}$	442.09	Flavanols
(-)-Epigallocatechin	$C_{15}H_{14}O_{7}$	306.07	Flavanols
(-)-Epicatechin	$C_{15}H_{14}O_{6}$	290.08	Flavanols
(-)-Catechin	$C_{15}H_{14}O_{6}$	290.08	Flavanols
Afzelechin	$C_{15}H_{14}O_{5}$	274.08	Flavanols
(-)-Gallocatechin gallate	$C_{22}H_{18}O_{11}$	458.08	Flavanols
Isosakuranin	$C_{22}H_{24}O_{10}$	448.14	Flavanones
Liquiritigenin	$C_{15}H_{12}O_{4}$	256.07	Flavanones
Isosakuranetin	$C_{16}H_{14}O_{5}$	286.08	Flavanones
Narirutin	$C_{27}H_{32}O_{14}$	580.18	Flavanones
Pinocembrin	$C_{15}H_{12}O_{4}$	256.07	Flavanones
Neohesperidin	$C_{28}H_{34}O_{15}$	610.19	Flavanones

Naringenin-7-glucoside	$C_{21}H_{22}O_{10}$	434.12	Flavanones
Sophoraflavanone G	$C_{25}H_{28}O_{6}$	424.19	Flavanones
Farrerol	$C_{17}H_{16}O_{5}$	300.10	Flavanones
Liquiritin	$C_{21}H_{22}O_{9}$	418.13	Flavanones
Hesperetin	$C_{16}H_{14}O_{6}$	302.08	Flavanones
Bavachin	$C_{20}H_{20}O_{4}$	324.14	Flavanones
Eriocitrin	$C_{27}H_{32}O_{15}$	596.17	Flavanones
Persicogenin	$C_{17}H_{16}O_{6}$	316.09	Flavanones
Alpinetin	$C_{16}H_{14}O_{4}$	270.09	Flavanones
Eriodictyol	$C_{15}H_{12}O_{6}$	288.06	Flavanones
Bavachinin	$C_{21}H_{22}O_{4}$	338.15	Flavanones
Hesperidin	$C_{28}H_{34}O_{15}$	610.19	Flavanones
Poncirin	$C_{28}H_{34}O_{14}$	594.19	Flavanones
Silibinin	$C_{25}H_{22}O_{10}$	482.12	Flavanonols
Taxifolin 7-O-rhamnoside	$C_{21}H_{22}O_{11}$	450.12	Flavanonols
Dihydrokaempferol	$C_{15}H_{12}O_{6}$	288.06	Flavanonols
Isosilybin	$C_{25}H_{22}O_{10}$	482.12	Flavanonols
Dihydromyricetin	$C_{15}H_{12}O_{8}$	320.05	Flavanonols
Astilbin	$C_{21}H_{22}O_{11}$	450.12	Flavanonols
Silychristin	$C_{25}H_{22}O_{10}$	482.12	Flavanonols
Engeletin	$C_{21}H_{22}O_{10}$	434.12	Flavanonols
Taxifolin	$C_{15}H_{12}O_{7}$	304.06	Flavanonols
Isoorientin	$C_{21}H_{20}O_{11}$	448.10	Flavone glycosides
Spinosin	$C_{28}H_{32}O_{15}$	608.17	Flavone glycosides
3'-Methoxypuerarin	$C_{22}H_{22}O_{10}$	446.12	Flavone glycosides
Vitexin	$C_{21}H_{20}O_{10}$	432.11	Flavone glycosides
Schaftoside	$C_{26}H_{28}O_{14}$	564.15	Flavone glycosides
Orientin	$C_{21}H_{20}O_{11}$	448.10	Flavone glycosides
Limocitrin	$C_{17}H_{14}O_{8}$	346.07	Flavones
Linarin	$C_{28}H_{32}O_{14}$	592.18	Flavones
Tangeretin	$C_{20}H_{20}O_{7}$	372.12	Flavones
IKarisoside A	$C_{26}H_{28}O_{10}$	500.17	Flavones
Apigenin-7-glucuronide	$C_{21}H_{18}O_{11}$	446.08	Flavones
Wogonin	$C_{16}H_{12}O_{5}$	284.07	Flavones
Scutellarein tetramethyl ether	$C_{19}H_{18}O_{6}$	342.11	Flavones
3,4'-Dihydroxyflavone	$C_{15}H_{10}O_{4}$	254.06	Flavones
Trimethylapigenin	$C_{18}H_{16}O_{5}$	312.10	Flavones
4',5-Dihydroxyflavone	$C_{15}H_{10}O_{4}$	254.06	Flavones
5-Hydroxyflavone	$C_{15}H_{10}O_{3}$	238.06	Flavones
Wogonoside	$C_{22}H_{20}O_{11}$	460.10	Flavones
Hispidulin	$C_{16}H_{12}O_{6}$	300.06	Flavones

Eupatorin	$C_{18}H_{16}O_7$	344.09	Flavones
Chrysosplenetin	$C_{19}H_{18}O_8$	374.10	Flavones
Baicalein	$C_{15}H_{10}O_5$	270.05	Flavones
6-Methylflavone	$C_{16}H_{12}O_2$	236.08	Flavones
6,2'-Dihydroxyflavone	$C_{15}H_{10}O_4$	254.06	Flavones
6-Hydroxyflavone	$C_{15}H_{10}O_3$	238.06	Flavones
Nobiletin	$C_{21}H_{22}O_8$	402.13	Flavones
Licoflavone A	$C_{20}H_{18}O_4$	322.12	Flavones
5,7,3',4'-Tetramethoxyflavone	$C_{19}H_{18}O_6$	342.11	Flavones
5-Methoxyflavone	$C_{16}H_{12}O_3$	252.08	Flavones
Sinensetin	$C_{20}H_{20}O_7$	372.12	Flavones
Tectochrysin	$C_{16}H_{12}O_4$	268.07	Flavones
5-O-Demethylnobiletin	$C_{20}H_{20}O_8$	388.12	Flavones
7,4'-Di-O-methylapigenin	$C_{17}H_{14}O_5$	298.08	Flavones
Acacetin	$C_{16}H_{12}O_5$	284.07	Flavones
7,4'-Dihydroxyflavone	$C_{15}H_{10}O_4$	254.06	Flavones
Cynaroside	$C_{21}H_{20}O_{11}$	448.10	Flavones
Sakuranetin	$C_{16}H_{14}O_5$	286.08	Flavones
Apigenin	$C_{15}H_{10}O_5$	270.05	Flavones
Luteolin	$C_{15}H_{10}O_6$	286.05	Flavones
Oroxin A	$C_{21}H_{20}O_{10}$	432.11	Flavones
Homoplantaginin	$C_{22}H_{22}O_{11}$	462.12	Flavones
Genkwanin	$C_{16}H_{12}O_5$	284.07	Flavones
5,7-Dihydroxy-3',4',5'-trimethoxyflavone	$C_{18}H_{16}O_7$	344.09	Flavones
Oroxin B	$C_{27}H_{30}O_{15}$	594.16	Flavones
hydroxygenkwanin	$C_{16}H_{12}O_6$	300.06	Flavones
Pedalitin	$C_{16}H_{12}O_7$	316.06	Flavones
Tricetin	$C_{15}H_{10}O_7$	302.04	Flavones
Scutellarin	$C_{21}H_{18}O_{12}$	462.08	Flavones
Tricin	$C_{17}H_{14}O_7$	330.07	Flavones
Chrysin	$C_{15}H_{10}O_4$	254.06	Flavones
Jaceosidin	$C_{17}H_{14}O_7$	330.07	Flavones
Scutellarein	$C_{15}H_{10}O_6$	286.05	Flavones
Baicalin	$C_{21}H_{18}O_{11}$	446.08	Flavones
Galangin	$C_{15}H_{10}O_5$	270.05	Flavones
Diosmin	$C_{28}H_{32}O_{15}$	608.17	Flavones
Diosmetin	$C_{16}H_{12}O_6$	300.06	Flavones
Apigenin 7-glucoside	$C_{21}H_{20}O_{10}$	432.11	Flavones
Nicotiflorin	$C_{27}H_{30}O_{15}$	594.16	Flavonols
Narcissin	$C_{28}H_{32}O_{16}$	624.17	Flavonols
Quercitrin	$C_{21}H_{20}O_{11}$	448.10	Flavonols

Sagittatoside A	$C_{33}H_{40}O_{15}$	676.24	Flavonols
Isorhamnetin	$C_{16}H_{12}O_7$	316.06	Flavonols
Rutin	$C_{27}H_{30}O_{16}$	610.15	Flavonols
Isorhamnetin-3-O-neohespeidoside	$C_{28}H_{32}O_{16}$	624.17	Flavonols
Kaempferol 3-neohesperidoside	$C_{27}H_{30}O_{15}$	594.16	Flavonols
3,7-Di-O-methylquercetin	$C_{17}H_{14}O_7$	330.07	Flavonols
Flavonol	$C_{15}H_{10}O_3$	238.06	Flavonols
Astragalin	$C_{21}H_{20}O_{11}$	448.10	Flavonols
Fisetin	$C_{15}H_{10}O_6$	286.05	Flavonols
Afzelin	$C_{21}H_{20}O_{10}$	432.11	Flavonols
Icariside I	$C_{27}H_{30}O_{11}$	530.18	Flavonols
Quercetin	$C_{15}H_{10}O_7$	302.04	Flavonols
Baimaside	$C_{27}H_{30}O_{17}$	626.15	Flavonols
3,7,4'-Trihydroxyflavone	$C_{15}H_{10}O_5$	270.05	Flavonols
Isorhamnetin 3-O-glucoside	$C_{22}H_{22}O_{12}$	478.11	Flavonols
Quercimeritrin	$C_{21}H_{20}O_{12}$	464.10	Flavonols
Hyperoside	$C_{21}H_{20}O_{12}$	464.10	Flavonols
Robinin	$C_{33}H_{40}O_{19}$	740.22	Flavonols
Kaempferitrin	$C_{27}H_{30}O_{14}$	578.16	Flavonols
Spiraeoside	$C_{21}H_{20}O_{12}$	464.10	Flavonols
Noricaritin	$C_{20}H_{20}O_7$	372.12	Flavonols
Myricetin	$C_{15}H_{10}O_8$	318.04	Flavonols
Miquelianin	$C_{21}H_{18}O_{13}$	478.07	Flavonols
Icaritin	$C_{21}H_{20}O_6$	368.13	Flavonols
Licoflavonol	$C_{20}H_{18}O_6$	354.11	Flavonols
Quercetin 3-O-(6''-galloyl)-β-D-galactopyranoside	$C_{28}H_{24}O_{16}$	616.11	Flavonols
Icariin	$C_{33}H_{40}O_{15}$	676.24	Flavonols
Baohuoside I	$C_{27}H_{30}O_{10}$	514.18	Flavonols
Tiliroside	$C_{30}H_{26}O_{13}$	594.14	Flavonols
Kaempferol	$C_{15}H_{10}O_6$	286.05	Flavonols
Myricitrin	$C_{21}H_{20}O_{12}$	464.10	Flavonols
2''-O-Galloylhyperin	$C_{28}H_{24}O_{16}$	616.11	Flavonols
Laricitrin	$C_{16}H_{12}O_8$	332.05	Flavonols
Kaempferide	$C_{16}H_{12}O_6$	300.06	Flavonols
Avicularin	$C_{20}H_{18}O_{11}$	434.08	Flavonols
Typhaneoside	$C_{34}H_{42}O_{20}$	770.23	Flavonols
Irisflorentin	$C_{20}H_{18}O_8$	386.10	Isoflavanones
Calycosin-7-O-β-D-glucoside	$C_{22}H_{22}O_{10}$	446.12	Isoflavanones
Calycosin	$C_{16}H_{12}O_5$	284.07	Isoflavanones
Formononetin	$C_{16}H_{12}O_4$	268.07	Isoflavanones

Neobavaisoflavone	$C_{20}H_{18}O_4$	322.12	Isoflavanones
7-Methoxyisoflavone	$C_{16}H_{12}O_3$	252.08	Isoflavanones
Tectorigenin	$C_{16}H_{12}O_6$	300.06	Isoflavanones
Licoisoflavone A	$C_{20}H_{18}O_6$	354.11	Isoflavanones
5-Methyl-7-methoxyisoflavone	$C_{17}H_{14}O_3$	266.09	Isoflavanones
Puerarin	$C_{21}H_{20}O_9$	416.11	Isoflavanones
Corylin	$C_{20}H_{16}O_4$	320.10	Isoflavanones
6''-O-Acetylglycitin	$C_{24}H_{24}O_{11}$	488.13	Isoflavanones
Daidzin	$C_{21}H_{20}O_9$	416.11	Isoflavanones
Demethyltexasin	$C_{15}H_{10}O_5$	270.05	Isoflavanones
2'-Hydroxydaidzein	$C_{15}H_{10}O_5$	270.05	Isoflavanones
Daidzein	$C_{15}H_{10}O_4$	254.06	Isoflavanones
Glabridin	$C_{20}H_{20}O_4$	324.14	Isoflavanones
Prunetin	$C_{16}H_{12}O_5$	284.07	Isoflavanones
Genistein	$C_{15}H_{10}O_5$	270.05	Isoflavanones
Genistin	$C_{21}H_{20}O_{10}$	432.11	Isoflavanones
Ononin	$C_{22}H_{22}O_9$	430.13	Isoflavanones
2'-Hydroxygenistein	$C_{15}H_{10}O_6$	286.05	Isoflavanones
Glycitin	$C_{22}H_{22}O_{10}$	446.12	Isoflavanones
Corylifol A	$C_{25}H_{26}O_4$	390.18	Isoflavanones
beta-Mangostin	$C_{25}H_{28}O_6$	424.19	Xanthones
Isomangiferin	$C_{19}H_{18}O_{11}$	422.08	Xanthones
Mangiferin	$C_{19}H_{18}O_{11}$	422.08	Xanthones
Procyanidin B2	$C_{30}H_{26}O_{12}$	578.14	Procyanidins
Kushenol A	$C_{25}H_{28}O_5$	408.19	Other flavonoids
Kurarinone	$C_{26}H_{30}O_6$	438.20	Other flavonoids
Mulberrin	$C_{25}H_{26}O_6$	422.17	Other flavonoids
Morusin	$C_{25}H_{24}O_6$	420.16	Other flavonoids
Methylnissolin-3-O-glucoside	$C_{23}H_{26}O_{10}$	462.15	Other flavonoids
Theaflavin	$C_{29}H_{24}O_{12}$	564.13	Other flavonoids
Hydroxysafflor yellow A	$C_{27}H_{32}O_{16}$	612.17	Other flavonoids
Epimedin A	$C_{39}H_{50}O_{20}$	838.29	Other flavonoids
Sciadopitysin	$C_{33}H_{24}O_{10}$	580.14	Other flavonoids
β-Anhydroicaritin	$C_{21}H_{20}O_6$	368.13	Other flavonoids
Epmedin C	$C_{39}H_{50}O_{19}$	822.29	Other flavonoids
Theaflavin 3,3'-digallate	$C_{43}H_{32}O_{20}$	868.15	Other flavonoids
Deguelin	$C_{23}H_{22}O_6$	394.14	Other flavonoids
Troxerutin	$C_{33}H_{42}O_{19}$	742.23	Other flavonoids
Epimedin B	$C_{38}H_{48}O_{19}$	808.28	Other flavonoids

3
