# Supplementary material for: Flavonoid Extract from Seed Residues of Hippophae rhamnoides ssp. sinensis Protects against Alcohol-Induced Intestinal Barrier Dysfunction by Regulating the Nrf2 Pathway
Source: Antioxidants (Basel). 2023 Feb 24;12(3):562. doi: 10.3390/antiox12030562 (PMC10044812; doi:10.3390/antiox12030562)
Supplement: Supplementary file 1 [file antioxidants-12-00562-s001.zip › antioxidants-2211060-supplementary-tableS2.pdf]

Table 1 Primer Sequence for quantitative reverse transcription polymerase chain reaction

genes	gene ID	forward (5’—3’)	reverse (5’—3’)	Product size (bp)
occludin	NM_002538.4	CCTCTTGAAAGTCCACCTC	GCCTACACTACCTCCTAAAA	297
zo-1	NM_003257.5	CCAGTCCCTTACCTTTCG	CTGCCTCATCATTTCCTC	264
Nrf2	NM_006164.5	CAGCGACGGAAAGAGTATGA	TGTGGGCAACCTGGGAGTAG	202
β-actin	NM_001101.5	GGCACCCAGCACAATGAA	TAGAAGCATTTGCGGTGG	168
